# Disease-related burden and treatment experience in patients with acromegaly: a gender perspective

**DOI:** 10.1007/s40618-026-02866-8

**Published:** 2026-04-15

**Authors:** Lilith Philomena Laflör, Sonja Siegel, Sabrina Giese, Jürgen Honegger, Anna Lena Friedel, Nicole Unger, Lukas van Baal, Karsten Henning Wrede, Ilonka Kreitschmann-Andermahr, Lisa Schock

**Affiliations:** 1 Department of Neurosurgery and Spine Surgery, Member of ENDO-ERN, University Hospital Essen, University of Duisburg-Essen, Essen, Germany; 2 Center for Translational Neuro- & Behavioral Sciences (C-TNBS), University of Duisburg- Essen, Essen, Germany; 3 German Cancer Consortium (DKTK) Partner Site, University Hospital Essen, University of Duisburg-Essen, Essen, Germany; 4 Department of Neurosurgery, Eberhard-Karls-University Tübingen, Tübingen, Germany; 5 Institute for Medical Education, University Hospital Essen, University of Duisburg-Essen, Essen, Germany; 6 Institute of Medical Psychology and Behavioral Immunobiology, University Hospital Essen, University of Duisburg-Essen, Essen, Germany; 7 Department of Endocrinology, Diabetes and Metabolism, Member of ENDO-ERN, University Hospital Essen, University of Duisburg-Essen, Essen, Germany; 8 Department of Neurosurgery, Community Hospital Mittelrhein, Koblenz, Germany

**Keywords:** Acromegaly, Gender differences, Comorbidities, Quality of life, Patient experience

## Abstract

**Objectives:**

Acromegaly, a rare endocrine disorder caused by excess growth hormone secretion, affects men and women similarly in prevalence, with women reporting a greater disease burden and a more impaired quality of life (QoL). It was the aim of the present study to investigate gender differences in subjective disease burden, comorbidities, treatment experience, satisfaction, and QoL in acromegaly, and assess whether QoL differences are mediated by comorbidities and/or gender-specific treatment experiences.

**Methods:**

In a multicenter cross-sectional study, 63 patients with biochemically confirmed acromegaly were surveyed using validated short scales (SF-12, WPAI, MK-HAI, ASKU, PDRQ-9) and self-developed questionnaires assessing sociodemographic data, disease status, subjective symptom load, comorbidities, and patient experiences. Statistical comparisons between men and women were conducted using nonparametric tests.

**Results:**

Women reported significantly higher symptom burden, musculoskeletal comorbidities, and health anxiety, alongside lower physical and mental QoL scores, compared to men. Women expressed lower satisfaction with therapy success and greater perceived treatment need, despite similar patient–doctor relationship quality ratings.

**Conclusions:**

Women with acromegaly experience a disproportionate physical and psychological burden relative to men. Our findings, including the subjective treatment experiences, point to the role of comorbidities and differences in treatment outcome in shaping this disparity and underscore the need for patient-centered, gender-sensitive approaches to acromegaly care.

**Supplementary Information:**

The online version contains supplementary material available at 10.1007/s40618-026-02866-8.

## Introduction

Gender medicine is the study of how diseases manifest differently in men and women and how therapies need to reflect these differences. For decades, biomedical research predominantly focused on the male physiology, with sex and gender differences rarely being assessed. More recently, growing evidence has demonstrated that both sex (the biological construct) and gender (the social construct) influence symptom manifestation, disease progression, treatment outcomes, and patient care across a broad spectrum of conditions [1].

Acromegaly, a rare chronic endocrine disorder caused by excessive secretion of growth hormone (GH), has recently come into the focus of gender-related research. Evidence suggests that women are more severely affected than men with higher prevalence of comorbidities and worse QoL, yet the underlying reasons remain unclear [2]. The disease is most often caused by a pituitary adenoma that leads to elevated levels of GH and insulin-like growth factor-1 (IGF-1), which drives its characteristic clinical features [3]. Patients typically present with disproportionate skeletal growth, soft tissue overgrowth, and coarse facial features [4, 5]. In addition to these physical changes, acromegaly is associated with a wide spectrum of comorbidities such as diabetes mellitus, sleep apnea, and musculoskeletal conditions [6, 7]. Though treatment options, including surgery, pharmacological therapies and radiotherapy have improved outcomes, acromegaly remains a challenging condition, which significantly impacts patients’ quality of life (QoL) and requires a multidisciplinary approach to management [8–10].

While prevalence is similar in women and men [11], evidence indicates that women consistently report a higher disease burden. Women experience greater impairments in both physical and mental aspects of QoL [12–16] and have a higher prevalence of associated mental health problems, including higher levels of depressive symptoms and anxiety [12–14, 17].

A review by Lenders et al. reports several indicators suggesting that the subjective burden experienced by women is due to a more severe course of the disease [2]. Women have a longer diagnostic delay and, in some studies, present with larger and more invasive tumors at diagnosis [18–20]. They also seem to suffer from hypertension and diabetes more often, albeit research results are conflicting in this regard [21, 22].

While the review [2] finds good treatment outcomes with normalized mortality in both men and women, women’s QoL appears to not improve as much as men’s, highlighting that more insight is needed into how comorbidities affect QoL in acromegaly and whether they might mediate the differential effect of the disease on QoL in both genders.

Moreover, nothing is known about gender-specific treatment experience, such as patients’ subjective interactions with the healthcare system and patient satisfaction, encompassing their expectations and subjective opinion on the care they received, in acromegaly yet. However, these aspects are crucial aspects, since evidence suggests, that there are significant differences in the care process between female and male patients, with physicians potentially making medical decisions based on gender-related factors [23]. However, it remains unclear whether such differences contribute to the less favorable outcomes in women with acromegaly.

Against this background, the present study investigates gender differences in subjective disease burden, comorbidities, patient experience and satisfaction as well as QoL. We aim to deepen the understanding of the extent to which gender influences the course of acromegaly and whether the difference in QoL in men and women is mediated by comorbidities and/or gender specific treatment experiences, thereby providing new insights for the development of gender-sensitive care strategies in this rare disease.

## Materials and methods

### Sample

This analysis is part of a cross-sectional, multicenter, non-interventional, postal questionnaire study by Siegel et al., which investigated influencing factors of adherence [24]. It was conducted at the Departments of Neurosurgery and Spine Surgery and Endocrinology, Diabetology and Metabolism at the University Hospital Essen and the Department of Neurosurgery, University Hospital Tübingen, Germany. The study used validated short scales as well as self-developed questionnaires to evaluate factors like condition-related, therapy-related and patient-related factors in acromegaly patients. For this study, we focused on adults with acromegaly. The sample comprised patients either actively engaged in aftercare at the participating clinics or those who had previously received treatment but were no longer attending follow-up visits. Eligibility criteria were presence of biochemically proven acromegaly due to a GH-secreting pituitary adenoma, sufficient command of the German language to complete a self-report questionnaire, as well as no active psychotic disorder. Patients who met the eligibility criteria were sent a study information sheet, a consent form and questionnaire package by mail.

Ethical approval for the study was granted by the ethics committees of the University of Essen (20-9447-BO) and the University of Tübingen and was conducted according to the Declaration of Helsinki. All patients included in the analysis provided written informed consent.

### Questionnaires

The following section provides an overview of the materials and methods relevant to this study.

### Self-developed questionnaires

The self-developed questionnaires used in this study covered sociodemographic information, current disease status, subjective symptom load, comorbidities, therapy course, doctor-patient communication and perceived therapy needs. The questionnaires included yes/no items, multiple-choice questions, free-text questions, and 5-point Likert-scale items (0 to 4). A mean score was calculated if several items measured the same construct. The complete questionnaires are available in the publication by Siegel et al. [24]. For the present analysis, selected scales from these questionnaires (perceived beneficial effects of therapy, perceived adverse effects of therapy, global impression of change, perceived therapy success, perceived need for treatment, usual duration of consultations, satisfaction with usual duration of consultations) were compared by gender. The detailed contents of the scales mentioned in this study can be found in the Supplementary Material.

### Validated short scales

#### 12-Item Short Form Health Survey (SF-12)

The SF-12 is a short tool designed to assess health-related QoL [25]. Comprising 12 items, the SF-12 provides separate measures for physical and mental aspects of QoL, resulting in the Physical Component Summary and the Mental Component Summary. The raw scores of the SF-12 can be converted into standardized T-values (*M* = 50.0, *SD* = 10.0), based on the normative data of the healthy population. A higher score reflects a better overall QoL and a T-value of 50 the healthy population mean.

#### Work Productivity and Activity Impairment (WPAI)

The WPAI questionnaire assesses the extent to which health struggles interfere with daily activities and work productivity with six items [26]. For non-employed individuals, only activity impairment is evaluated. The results are expressed as a percentage, indicating the proportion of time affected by impairment.

#### Modified Short Form Health Anxiety Inventory (Modifizierte Kurzform des Health Anxiety Inventorys, MK-HAI)

The MK-HAI is a validated short scale designed to assess patients’ tendencies toward health-related worries [27]. Adapted from the Health Anxiety Inventory [28], it comprises 14 statements addressing health anxiety over the past six months. Patients rate their agreement with each statement on a five-point Likert scale (0 to 4), resulting in a total score. Higher scores indicate greater levels of health anxiety.

#### General Self-Efficacy Scale (Allgemeine Selbstwirksamkeit Kurzskala, ASKU)

The ASKU evaluates individuals’ perceived ability to handle daily difficulties in a concise three-item instrument [29]. Participants respond on a five-point Likert scale (1 to 5), with higher ratings indicating stronger self-efficacy. Reference data segmented by age and gender are accessible for the German population, allowing for contextual interpretation of the results.

#### Patient-Doctor Relationship Questionnaire (PDRQ-9)

The PDRQ-9 is a nine-item tool used to evaluate the quality of the doctor-patient relationship [30]. Patients rate their agreement with statements about their physician on a five-point Likert scale (1 to 5), resulting in an overall mean score. A higher average score reflects a stronger and more positive doctor-patient alliance.

### Statistical analysis

Statistical analyses were performed using SPSS (IBM Corp. Released 2023. IBM SPSS Statistics for Windows, Version 29.0.2.0 Armonk, NY: IBM Corp.). Interval-scaled variables were summarized as mean (M) and standard deviation (SD) for the total sample. For subgroup analyses, medians are reported to account for potential distortions in smaller groups. Categorical variables were expressed as frequencies and valid percentages. Comparisons of interval-scaled data between genders (female, male) were conducted using the Mann–Whitney U test, as data distributions were not assumed to be normal. Z-values are reported to provide a standardized measure of the observed rank differences. Chi-square tests or Fisher’s exact tests, where appropriate, were applied to analyze differences in categorical variables. Correlation analyses were performed using Spearman’s Rho. Missing data were handled using casewise deletion, and statistical significance was set at an alpha level of *p* < .05. Due to the exploratory nature of the analysis, no corrections for multiple testing were applied to reduce the risk of beta errors.

## Results

### Demographic and clinical characteristics

Table 1 provides an overview of the sociodemographic and clinical characteristics of the study sample broken down by gender. Men and women did not significantly differ in age (*t* (61) = -1.28, *p* = .204; Table 1). Men (48.3%, *n* = 14) held a university degree more often than women (14.8%, *n* = 4) (*χ*² (3) = 7.18, *p* = .007) and were more frequently employed (men: 76.7%, *n* = 23 vs. women: 30.0%, *n* = 9; *χ*² (5) = 16.83, *p* = .005). Notably, 40.0% of women (*n* = 12) were retired, compared to only 16.7% of men (*n* = 5).

Men and women did not differ regarding their received therapies. 100% of men (*n* = 30) and 93.9% of women (*n* = 31) underwent pituitary surgery and 16.7% of men (*n* = 5) and 9.1% of women (*n* = 3) received radiation therapy, (*χ*² (1) = 1.88, *p* = .171; *χ*² (1) = 0.81, *p* = .367). There was no difference with regard to disease activity, with 83.3% of men (*n* = 20) and 87.5% (*n* = 21) of women reporting normalized IGF-1 at the last examination (*χ*² (1) = 0.17, *p* = .683).

**Table 1:** Sociodemographic and clinical characteristics of men and women in the study sample

Category	Men		Women		*p*-value
	M (SD)	*n* (%)	M (SD)	*n* (%)	
**Participants**		30 (47.6)		33 (52.4)	
**Age**	53.67 (13.80)		58.24 (14.44)		0.20
**Time since diagnosis (years)**	12.92 (9.20)		11.16 (7.71)		0.43
**BMI (Body Mass Index)**	29.11 (3.93)		28.58 (6.28)		0.69
**Working Situation**					**0.01**
Full time		20 (66.7)		5 (15.2)	
Part time		3 (10.0)		4 (12.1)	
Disability pension		1 (3.3)		3 (9.1)	
Retired/ not working for other reason		6 (20.0)		18 (54.5)	
**Normalized IGF-1 (self-reported)**		20 (66.7)		21 (63.6)	0.68
**Acromegaly-Specific Medication**					
Somatostatin analogues		10 (33.3)		13 (39.3)	0.62
Dopamine agonists		2 (6.7)		1 (3.0)	0.46^a^
GH-receptor antagonists		4 (13.3)		10 (30.3)	0.11
**Substitution of Hormonal Deficiencies**					
gonadotropic		2 (6.7)		0 (0.0)	0.22^a^
thyrotropic		4 (13.3)		10 (30.3)	0.11
corticotropic		0 (0.0)		4 (12.1)	0.07^a^
posterior pituitary		0 (0.0)		4 (12.1)	0.07^a^
**Other Medication**					
Antidepressants		0 (0.0)		4 (12.1)	0.07^a^
Antihypertensives		10 (33.3)		17 (51.5)	0.15
Antidiabetics		1 (3.3)		6 (18.2)	0.07^a^
Analgesics		2 (6.7)		11 (33.3)	**0.01**

This table presents the sociodemographic and clinical characteristics of the study participants, divided by male and female, with averages or absolute frequencies (percentages) reported. ^a^ Fisher’s exact test

### Physical impairment

**Fig. 1:**
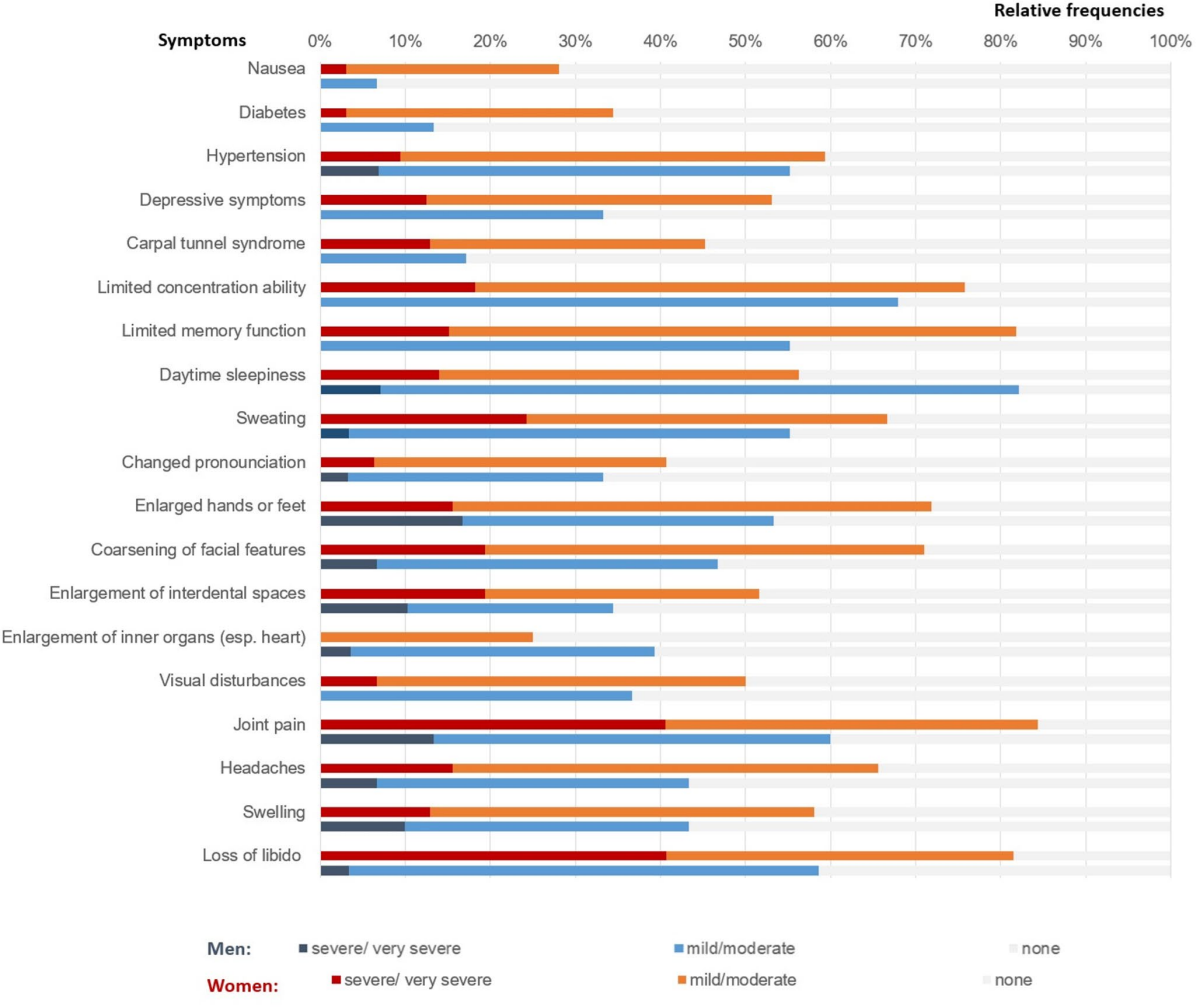
Gender differences in the severity of self-reported physical symptoms in acromegaly. Relative frequencies of symptom severity (none, mild/moderate, severe/very severe) are displayed for men and women

The subjective symptom load of acromegaly was significantly higher in women (*Mdn* = 39.09) compared to men (*Mdn* = 24.20; *U* = 729.0, *z* = 3.221, *p* = .001; confer Fig. 1).

**Fig. 2:**
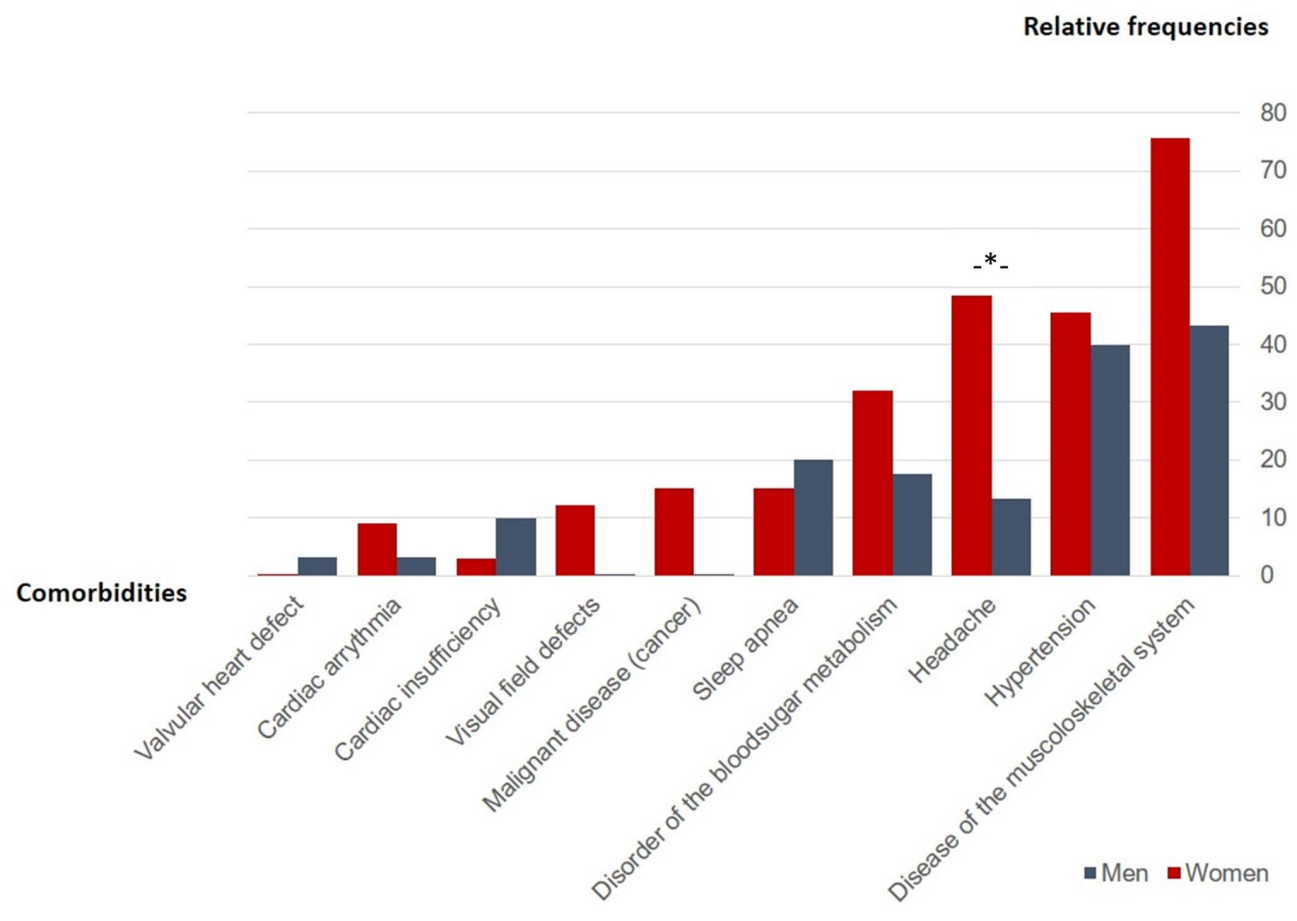
Gender differences in comorbidities among patients with acromegaly. Relative frequencies of self-reported comorbidities by gender. * *p* < .05

Moreover, 30.3% of women suffered from serious or life-threatening comorbidities^1^ compared to 16.7% of men (Fisher’s exact test *p* = .003). Most notably, 76% of women, but only 43% of men suffered from musculoskeletal disease and only 13% of men reported headaches, compared to 49% of women (confer Fig. 2).

Women (*Mdn* = 36.08) also reported significantly higher activity impairment due to health on the WPAI than men (*Mdn* = 22.29, *U* = 629.5, *z* = 3.121, *p* = .002) and held a recognized degree of disability almost twice as often as men (women: 62.5%, *n* = 20 vs. men: 34.5%, *n* = 10; χ² (1) = 4.78, *p* = .029). In line with the more severe physical impairment in all measures reported above, women (*Mdn* = 23.24) had significantly lower scores on the physical sum scale of the SF-12 than men (*Mdn* = 37.48, *U* = 224.5, *z* = -3.180, *p* = .001), indicating a lower physical QoL in women.

### Mental impairment

Men (*Mdn* = 34.91) had higher scores on the mental sum scale of the SF-12 than women (*Mdn* = 25.56; *U* = 296.5, *z* = -2.087, *p* = .037), indicating that women experienced a significantly poorer mental QoL than the male study participants. Women (*Mdn* = 35.80) also had a higher degree of health anxiety as measured by the MK-HAI compared to men (*Mdn* = 24.45; *U* = 617.5, *z* = 2.513, *p* = .012). There was no significant difference in the general self-efficacy expectations measured by the ASKU between men (*Mdn* = 35.59) and women (*Mdn* = 27.91; *U* = 360.0, *z* = -1.706, *p* = .088) implying a similar level of confidence in their ability to cope with their condition.

### Patient experience and patient satisfaction

There were no significant gender differences in the perceived beneficial effects of therapy. Men (*Mdn* = 27.37) and women (*Mdn* = 26.65) reported similar improvements in managing daily life (*U* = 341.5, *z* = -0.170, *p* = .865), with 62% of men and 63% of women indicating that they could handle daily life better than before therapy. Likewise, perceived adverse effects of therapy did not differ significantly between men (*Mdn* = 23.94) and women (*Mdn* = 29.06; *U* = 404.5, *z* = 1.225, *p* = .221), with 26.1% of men and 30.4% of women reporting to suffer from medication side effects. Similarly, no significant differences emerged in the global impression of change in physical well-being (men: *Mdn* = 31.30; women: *Mdn* = 26.93; *U* = 343.0, *z* = -1.039, *p* = .299) or mental well-being (men: *Mdn* = 28.85; women: *Mdn* = 29.13; *U* = 409.0, *z* = 0.068, *p* = .946) since the beginning of treatment. Overall, 55.5% of men and 53.4% of women reported feeling physically a little or much better, while 37% of men and 53% of women indicated improvements in mental well-being. However, perceived therapy success was lower among women (*Mdn* = 25.09) than men (*Mdn* = 33.05), with 89% of men and 65.5% of women rating the therapy as successful or very successful. The difference approached statistical significance (*U* = 292.5, *z* = -1.937, *p* = .053). In contrast, the perceived need for treatment was significantly higher in women (*Mdn* = 33.78) than in men (*Mdn* = 25.22; *U* = 544.5, *z* = 2.004, *p* = .045). 41% of men and only 7% of women believed they had little or no need for treatment.

With regard to the patient-doctor relationship as measured by the PDRQ-9, no significant difference was observed between men (*Mdn* = 35.64) and women (*Mdn* = 27.86; *U* = 358.5, *z* = -1.705, *p* = .088). The usual duration of consultations during routine appointments was below ten minutes for 49% of women and 31% of men. However, the difference was not significant (*χ²*(1) = 1.95, *p* = .162). 72.4% of men and 75.8% of women perceived the usual duration of a routine appointment as exactly right (*χ²*(2) = 0.09, *p* = .956).

## Discussion

The present study aimed to advance understanding of gender differences in acromegaly by examining not only subjective disease burden and comorbidities but also treatment experiences, satisfaction and QoL. In line with previous reports [2, 12–14], our results confirm that women living with acromegaly experience a significantly higher disease burden than men despite comparable disease duration, overall treatment modalities, and self-reported biochemical control. Importantly, our findings extend prior knowledge by highlighting that comorbidities and subjective treatment perceptions are central in shaping this gender disparity.

Women in our study reported substantially higher rates of musculoskeletal disorders and headaches, alongside a greater overall comorbidity burden. These somatic conditions are reflected in lower scores on the physical component score of the SF‑12 and a higher prevalence of recognized disability status as well as higher intake of analgesics, compared to their male counterparts. Taken together, this suggests that comorbidities act as critical mediators of poor physical QoL in women. This observation complements the findings of Lenders et al. [2], who also identified diagnostic delay, tumor invasiveness, and comorbidities as potential explanations for the more severe course of disease in women. Notably, seven female participants – compared to only three men – were receiving GH receptor agonist therapy, a second-line medical treatment typically reserved for patients with inadequate response to first-generation somatostatin receptor ligands. This disparity in treatment intensity supports the notion that women in our cohort experienced a more severe disease manifestation, possibly stemming from delayed diagnosis. All in all, our results reinforce the notion that effective management of comorbidities — particularly musculoskeletal disease and pain — could be key to improving patient-reported outcomes in female patients.

Mental health burden was likewise more pronounced in women, who reported poorer mental QoL, higher health-related anxiety, and less perceived benefit from treatment. These findings resonate with prior studies documenting increased self-reported depressive symptoms and anxiety rates in women with acromegaly [13, 17]. A key contributing factor could be the greater psychological distress women experience due to the visible physical changes caused by the disease, which negatively affect body image and self-esteem [13, 31, 32]. Interestingly, general self-efficacy did not differ by gender, suggesting that women do not feel less able to cope with their disease per se, but may be systematically disadvantaged by the greater symptomatic load and its psychosocial consequences.

Regarding patient experience, our results provide first insights into an area previously unexplored in acromegaly research. Men and women evaluated their patient–doctor relationship similarly, and both reported comparable benefits and side effects of therapy. Nevertheless, women rated their treatment as less successful and expressed a higher ongoing need for care. This is most likely a consequence of the longer diagnostic delay in women [2, 20, 33, 34], causing a higher symptom load and more comorbidities, placing women with acromegaly at a disadvantage from the start. The reasons for this gender bias should be further elucidated in future investigations, considering explanations such as a greater difficulty to recognize the disease in women due to higher variety of symptoms [33, 34]. Despite reporting a comparable impression of change due to therapy, in our study, women with acromegaly did not achieve the same functional level as men. These findings shed new light on the interpretation of consistently poorer QoL and higher psychological burden among women with acromegaly: It is not merely that women complain more; rather, they carry a heavier disease burden at diagnosis and even after successful treatment.

These results have important implications. First, they confirm, that biochemical remission should not be viewed as the sole endpoint in acromegaly care. Incorporating patient-reported outcomes into regular follow-up is essential, particularly for female patients whose disease burden may be underestimated if evaluation is limited to hormonal markers. Second, gender-sensitive care strategies should prioritize comorbidity management and structured psychosocial support to address both physical and mental health burden in women with acromegaly. Finally, physicians should be aware that subjective treatment satisfaction may differ by gender and should actively inquire about expectations and unmet needs in clinical consultations.

Our study had some limitations, that must be acknowledged. The cross-sectional design precludes conclusions about cause and effect or the direction of relationships between gender, comorbidities, treatment satisfaction, and QoL. For the classification of biochemical control, self-reported IGF-1 levels were relied on, as was the case for the classification of hormone replacement therapy, which is susceptible to error. The sample size was modest, limiting statistical power and subgroup analyses [35], therefore we refrained from conducting covariance analysis to adjust for potential confounders. Due to the lack of short scales with gender-adjusted normative values for the current research question, self-developed instruments were applied. We are well aware that self-report studies are subject to potential bias. However, we have previously emphasized the importance of measuring patient experience and satisfaction [36].

The difference in education and income/working status, which is also documented in the literature [37], may have influenced disease management, such as health literacy, self-management strategies, and access to supportive resources, thereby affecting well-being. However, we emphasize that the observed gender differences were consistent across multiple outcome domains and considerable in magnitude. This makes it unlikely that they can be fully explained by baseline socioeconomic differences alone.

Because of the rarity of the disease, it was not possible to investigate gender other than in a binary manner, which does not capture the full spectrum of sex and gender diversity emphasized in contemporary gender medicine.

In conclusion, this study strengthens the evidence that women with acromegaly experience a higher disease burden and poorer physical and mental QoL than men. Our findings, including the subjective treatment experiences, point to the role of comorbidities and differences in treatment outcome in shaping this disparity and underscore the need for patient-centered, gender-sensitive approaches to acromegaly care. Addressing these dimensions could help reduce gender inequities, improve QoL, and guide more comprehensive treatment strategies in this rare condition.

## Supplementary Information

Below is the link to the electronic supplementary material.

Supplementary Material 1

## Data Availability

Any data not published within the article will be shared in an anonymized manner at request from any qualified investigator.

## Footnotes

1 Defined as in Siegel et al. (2024): Common comorbidities: hypertension, headache, sleep apnea; severe comorbidities: muscoskeletal disease, visual field defects, diabetes; potentially life-threatening comorbidities: cardiac disease, malignant disease.
