# Supplementary material for: Disease-related burden and treatment experience in patients with acromegaly: a gender perspective
Source: J Endocrinol Invest. 2026 Apr 15;49(8):1841–9. doi: 10.1007/s40618-026-02866-8 (PMC13486022; doi:10.1007/s40618-026-02866-8)
Supplement: Supplementary file 1 [file 40618_2026_2866_MOESM1_ESM.docx]

**Supplement 1**

**Patient experience and patient satisfaction items and scales from the self-developed questionnaire**

**Perceived beneficial effects of therapy**

What specific changes have you noticed since you started therapy?

- I can accept the physical changes caused by acromegaly.
- I am more open about my disease.
- I can manage my everyday life better again.
- I understand the disease and the changes it brings or has brought.

(fully agree, rather agree, neither nor, rather not agree, not agree at all)

**Perceived adverse effects of therapy**

What specific changes have you noticed since you started therapy?

- I feel stressed by the doctor's appointments.
- Therapy takes up a lot of space in my everyday life.
- I am burdened by taking the medication every day.
- I suffer from the side effects of the medication.

(fully agree, rather agree, neither nor, rather not agree, not agree at all)

**Global impression of change**

Has your physical or mental (psychological) condition changed since you started therapy?

- My physical condition since I started therapy has been ...
- My mental (psychological) condition since I started therapy has been ...

(much better, better, unchanged, worsened, strongly worsened)

**Perceived therapy success**

Overall, how successful do you experience your therapy?

(very successful, successful, moderate, of little success, not successful at all)

**Perceived need for treatment**

What is your own estimation of the need for medical therapy in regard to your acromegaly disease?

(no need, low demand, average demand, high demand, very high demand)

**Usual duration of consultations**

How long does it usually take to talk to your doctor at a routine appointment?

(less than 5 minutes, between 5 and 10 minutes, between 10 and 20 minutes, more than 20 minutes)

**Satisfaction with usual duration of consultations**

Do you feel that this duration is appropriate?

(far too long, a little too long, just right, a little too short, far too short)
